# Pre-Diagnosis Metabolic Health-Related Dietary Index and Prostate Cancer–Specific Mortality

**DOI:** 10.3390/nu18213703

**Published:** 2026-11-08

**Authors:** Sithembiso S. Msibi, Kidist Woldehawariat, Nguyet Trinh, MinJae Lee, Lihchyun Joseph Su

**Affiliations:** 1 Peter O’Donnell School of Public Health, UT Southwestern Medical Center, Dallas, TX 75390, USA; 2 Division of Clinical & Translational Sciences, McGovern Medical School, UTHealth, Houston, TX 77030, USA; minjae.lee@uth.tmc.edu

**Keywords:** prostate cancer, prostate cancer-specific mortality, metabolic health-related dietary index

## Abstract

**Background:** Metabolic dysfunction has been linked to prostate cancer (PCa) development and progression. This study sought to assess whether metabolic risk factors influence PCa death or simply reflect overall mortality risk among men with PCa. **Methods:** We analyzed data from 1497 men enrolled in the North Carolina-Louisiana Prostate Cancer Project (PCaP). Pre-diagnosis dietary and metabolic factors were summarized into a standardized metabolic health-related (MHD) index reflecting higher intake of added sugars, glycemic load, saturated fat, and sodium intake, and lower intake of fiber, fruit, vegetable, and whole grain. Adjusting for demographic, clinical and lifestyle factors, we used multinomial logistic regression to examine associations between the metabolic index and outcomes: PCa death, non-PCa death, and alive at follow-up. **Results:** Among 1497 men, 95 and 311 died from PCa and non-PCa causes, respectively, and 1091 were alive at follow-up. The MHD index differed across mortality-status groups (*p* < 0.001) and was higher among men with aggressive PCa (*p* < 0.001) but did not differ significantly by race (*p* = 0.187). In adjusted multinomial logistic models, higher MHD index, which represents a less favorable metabolic health–related dietary profile, was significantly associated with increased odds of PCa-specific death adjusted odds ratio [aOR] = 1.38 (95% CI, 1.10–1.73; *p* = 0.006), but not non-PCa death [aOR] = 1.03 (95% CI, 0.89–1.19; *p* = 0.687). **Conclusions:** Less favorable pre-diagnosis metabolic health–related dietary profiles were significantly associated with PCa-specific mortality but not non-PCa mortality, suggesting a potentially distinct role of dietary pattern in PCa progression. The association was not observed for non-PCa death and was attenuated after adjustment for anthropometric and tumor characteristics. These findings support further investigation of overall dietary-metabolic profiles as potentially relevant factors in PCa-specific mortality.

## 1. Introduction

With the exception of nonmelanoma skin cancers, prostate cancer (PCa) is the most often diagnosed cancer in men in the US and continues to be the second most common cause of cancer-related deaths among American men [1]. Despite its substantial disease burden, PCa generally has a favorable prognosis, with a 5-year relative survival rate of approximately 98% across all stages combined and greater than 99% for localized or regional disease [2]. Consequently, many men diagnosed with PCa live for extended periods and may ultimately die from causes other than their cancer, particularly given the older age at which PCa is commonly diagnosed and its often-prolonged clinical course. Therefore, it is crucial to differentiate factors associated with PCa-specific mortality from those associated with competing causes of death. Metabolic health may be particularly relevant in this context, although previous studies have reported differing associations of metabolic factors with PCa-specific and non-PCa mortality [3].

The progression and mortality of PCa have also been linked to several metabolic disorders. Through mechanisms involving insulin and insulin-like growth factor signaling, inflammation, lipid metabolism, and altered energy balance, hyperinsulinemia, insulin resistance, obesity, diabetes, and metabolic syndrome have been suggested as possible contributors to the advancement of PCa. Metabolic problems during midlife have been linked to a higher risk of eventually dying from PCa [4], while prospective studies have also associated high insulin levels with lethal PCa [5]. Recently, an analysis of eight European cohorts looked at the triglyceride-glucose index and body mass index as indicators of metabolic dysfunction in relation to prostate cancer mortality and there was no clear correlation found between the triglyceride-glucose index and PCa mortality [6]. These studies point to a possible connection between metabolic dysfunction and clinically significant PCa outcomes, but they also show that relationships differ depending on the metabolic exposure under investigation.

Dietary patterns characterized by high consumption of refined carbohydrates, added sugars, saturated fats, and other components of Western dietary patterns have been associated with adverse cardiometabolic outcomes [7,8]. Conversely, diets rich in fruits, vegetables, fiber, and whole grains may improve metabolic regulation and reduce inflammatory and insulin-related signaling [9]. Previous epidemiologic studies have suggested that Western, pro-inflammatory, and hyper-insulinemic dietary patterns may be associated with PCa progression or mortality, although findings have not been consistent across studies [10,11]. A more hyper-insulinemic eating pattern was linked to higher chances of advanced and deadly PCa in the Health Professionals Follow-up Study [12]. Additionally, post-diagnostic inflammatory, hyper-insulinemic, and insulin-resistant dietary and lifestyle behaviors were similarly linked to an increased risk of disease progression in males with nonmetastatic PCa [13]. Nevertheless, there was no clear correlation between these dietary patterns and PCa-specific mortality. Similarly, a follow-up analysis did not find any significant correlation between post-diagnostic inflammatory or insulinemic dietary and lifestyle indices and PCa-specific mortality [14]. Therefore, it is unclear how metabolically unfavorable eating habits relate to death from PCa specifically, even though they may be linked to aggressive or progressive disease.

Additional evidence that diet prior to diagnosis is associated with aggressive prostate cancer comes from studies of individual dietary factors and broader dietary patterns. In the North Carolina–Louisiana Prostate Cancer Project (PCaP), higher saturated fat intake was associated with higher odds of high-aggressive prostate cancer and higher dietary fiber intake was inversely associated with prostate cancer aggressiveness [15,16]. Comparable observations have been reported in other prospective cohorts. Higher saturated fat intake was related to increased risks of advanced and fatal prostate cancer in the NIH-AARP Diet and Health Study [17]. Furthermore, greater grain fiber intake was related to lower risks of advanced prostate cancer and prostate cancer mortality in a pooled analysis of 15 prospective cohorts [18]. Higher adherence to a Mediterranean dietary pattern was also associated with lower odds of high-aggressive prostate cancer in the PCaP study [19] in addition to individual nutrients. Evidence for carbohydrate-related exposures is less extensive. But a recent target trial emulation suggested that limiting sugar-sweetened beverage intake was associated with modestly lower risks of lethal and fatal prostate cancer [20]. These studies suggest that several dietary features associated with metabolic health could be associated with aggressive or fatal prostate cancer, although the findings have not been consistent across exposures or study populations. We hypothesized that a less favorable pre-diagnosis metabolic health–related dietary profile would be associated with PCa-specific mortality. Therefore, we examined associations between a pre-diagnosis metabolic health–related dietary index and cause-specific mortality among men with PCa in the PCaP study.

## 2. Materials and Methods

### 2.1. Study Population

The PCaP study recruited and enrolled residents of North Carolina (NC) and Louisiana (LA) study areas. To be eligible for the study, the participants must have had a first diagnosis of histologically confirmed adenocarcinoma of the prostate [21,22]. Participants were 40–79 years old and were diagnosed on or after 1 July 2004. Further, eligible participants could complete the interview in English and self-identified as African American (AA) or European American (EA). Cases in nursing homes, with severe cognitive or physical impairments, or under the influence of substances were excluded. Physician permission was required for contact. NC had lower response rates (38.1%) compared to LA (48.8%). A total of 2258 men enrolled in the PCaP (1130 AAs and 1128 EAs). The analytic population comprised 1497 PCaP study participants (722 AA and 775 EAs) with complete data on PCa tumor aggressiveness. Participants with missing data required for the multivariable models were excluded from the respective complete-case analyses. Written informed consent was obtained from all participants prior to data collection and the use of de-identified data for analysis and publication. The study protocol was approved by the Institutional Review Boards (IRB) of the University of North Carolina at Chapel Hill, Louisiana State University Health Sciences Center, and the Department of Defense Prostate Cancer Research Program (approval code: NHD 03262023, approval date: 26 March 2023). Additional IRB review was obtained at the University of Texas Southwestern Medical Center for the secondary data analysis for this manuscript.

### 2.2. Prostate Cancer Aggressiveness and Mortality Outcomes

The histologic stage of the PCa diagnosis was determined by the study pathologist through an independent review of diagnostic samples when available; otherwise, the data were abstracted directly from medical records. Pathologic stage, grade, and additional prostatectomy information were separately recorded when accessible. PCa cases were categorized based on Gleason grade, histologic stage and PSA levels. High aggressiveness (Gleason sum ≥ 8 or PSA > 20 ng/mL, OR Gleason sum = 7 with stage T3–T4), low aggressiveness (Gleason sum < 7, stage T1–T2, and PSA < 10 ng/mL) and intermediate aggressiveness which are all the other cases not fitting the high or low category [22]. Because the intermediate-aggressiveness category was defined by exclusion rather than by a distinct set of clinical criteria, it comprised a heterogeneous group with limited clinical interpretability. We therefore excluded these cases to provide a clearer comparison between the clinically well-defined high- and low-aggressiveness groups. A total of 1497 participants were included with high (n = 396) and low (1101) aggressive PCa in this analysis. Mortality was ascertained through linkage with the Louisiana Tumor Registry for Louisiana participants and North Carolina Vital Records for North Carolina participants, with follow-up extending through 31 March 2020 [23]. These sources provided information on vital status and causes of death. However, the de-identified dataset available for the present analysis did not include individual dates of death or follow-up dates, preventing the calculation of participant-specific follow-up durations and the use of time-to-event survival models. Accordingly, multinomial logistic regression was employed to investigate whether the pre-diagnosis metabolic health-related dietary (MHD) index was associated with odds of PCa-specific death or death from other causes, relative to being alive at follow-up, after adjustment for potential confounders. The primary outcome was mortality status classified into three mutually exclusive categories, namely, (1) alive, (2) death due to PCa and (3) death due to causes other than PCa. Alive participants served as the reference outcome in multinomial regression models.

### 2.3. Assessment of Metabolic Health-Related Dietary Index

Dietary factors were assessed using a modified National Cancer Institute (NCI) Diet History Questionnaire (DHQ) [24]. The 144-item questionnaire assessed the frequency of consumption, usual portion size, and food preparation practices during the 12 months preceding PCa diagnosis. For this analysis, we used an investigator-defined, study-specific index. Eight dietary components were selected a priori before examination of their associations with mortality outcomes. The components represent complementary dimensions of diet relevant to metabolic health, including glycemic burden (added sugars and glycemic load), dietary fat quality (saturated fat), sodium intake, carbohydrate quality (dietary fiber and whole grains), and plant-food intake (fruit and vegetables). Component selection and scoring direction were informed by established diet-quality indices and literature linking these dietary characteristics with cardiometabolic, insulinemic, and inflammatory pathways [25,26]. Established indices such as the Healthy Eating Index and Alternate Healthy Eating Index similarly favor greater intake of fruits, vegetables, and whole grains and lower intake of added sugars, sodium, and adverse dietary fats.

Since the dietary components were measured on different scales, each component was standardized to a z-score by subtracting the study-population mean from the participant-specific value and dividing by the corresponding standard deviation, consistent with approaches used in the construction of composite dietary indices [27,28]. Added sugars, glycemic load, percentage of energy from saturated fat, and sodium were positively scored, whereas dietary fiber, fruit, vegetables, and whole grains were negatively scored. Each component was standardized to a z-score, and components were assigned equal weights because there no component was assumed to be a priori to be more important than the other and giving differential weights to components would require an established empirical basis. Higher scores represented a more adverse dietary-metabolic profile. Table S1 shows the dietary components and scoring parameters used to construct the metabolic health-related dietary (MHD) index. After combining the eight standardized dietary components, the resulting composite MHD index was further standardized by subtracting its mean and dividing by its standard deviation in the analytic population (n = 1497). The standard deviation of the original composite index was approximately 2.90. This standardized composite score was used as the continuous exposure variable in the regression analyses. Accordingly, the reported adjusted odds ratios represent associations per one-standard-deviation increase in the overall MHD index, equivalent to approximately 2.90 units of the original composite score.

Each dietary component was standardized as follows:

$$Z=\frac{X_{ij\;}-{\bar{X}}_{j}}{{SD}_{j}}$$

where:

$Z_{ij}$ = standardized score for dietary component j for participant i.

$X_{ij}$ = participant i’s observed intake of component j.

${\bar{X}}_{j}$ = mean intake of component j in the analytic population.

$SD_{j}$ = standard deviation of component j in the analytic population.

For example, for sodium:

$$Z_{\mathrm{sodium},i}=\frac{{\mathrm{participant}\;\mathrm{sodium}\;\mathrm{intake}}_{i}-global\mathrm{mean}\;\mathrm{sodium}\;\mathrm{intake}}{\mathrm{global}\;\mathrm{SD}\;\mathrm{of}\;\mathrm{sodium}\;\mathrm{intake}}$$

Then the MHD index was calculated as:

$$\begin{array}{cc} {\mathrm{MHD}\;\mathrm{Index}}_{i}= & Z_{\mathrm{Added}\;\mathrm{Sugars}}+Z_{\mathrm{Glycemic}\;\mathrm{Load}}+Z_{\mathrm{Saturated}\;\mathrm{Fat}}+Z_{\mathrm{Sodium}}\\ & -Z_{\mathrm{Fiber}}-Z_{\mathrm{Fruit}}-Z_{\mathrm{Vegetables}}-Z_{\mathrm{Whole}\;\mathrm{Grains}} \end{array}$$

### 2.4. Statistical Analysis

Participant characteristics were summarized overall and according to mortality status. Continuous variables were summarized using means and standard deviations or medians and interquartile ranges, as appropriate, and categorical variables using frequencies and percentages. Differences in MHD index distributions across the three mortality categories were evaluated using the Kruskal–Wallis test. Differences according to race and tumor aggressiveness were evaluated using Wilcoxon rank-sum tests. Additionally, the MHD index was categorized into tertiles.

Multinomial logistic regression was used to estimate odds ratios (ORs) and 95% confidence intervals (CIs) for non-PCa death and PCa death, with alive at follow-up as the reference outcome. The MHD index was modeled continuously, with the OR representing the association per 1-SD higher index score. Multivariable analyses were conducted using complete-case analysis. Of the 1497 participants in the analytic sample, 1486 had complete data for the exposure, mortality outcome, and covariates included in the fully adjusted model. This is shown in Figure 1. Continuous and categorical MHD index models were fit using this same complete-case analytic sample. Sequential models were examined to assess the stability of the index association with increasing covariate adjustment. The primary multivariable multinomial logistic regression model adjusted for age at diagnosis, race, study site, smoking status, insurance status, previous screening and waist-to-hip ratio (WHR). For additional sensitivity analysis, we added tumor aggressiveness as an additional adjustment because it was assessed at diagnosis and could potentially represent a downstream characteristic of pre-diagnosis dietary exposure. We added tumor aggressiveness in the sensitivity model to evaluate whether associations were robust to differences in disease severity at diagnosis. The primary model was used for the main interpretation of the findings, while the aggressiveness-adjusted model was considered supplementary and used to assess the sensitivity of the estimates to additional adjustment for clinical disease characteristics.

To evaluate trends across tertiles of the MHD index, participants were assigned the median standardized MHD index value of their respective tertile. These tertile-specific median values were modeled as a continuous variable in the adjusted multinomial logistic regression model. Two-sided *p*-values for trend were calculated using Wald tests of the corresponding regression co-efficients, with being alive at follow-up serving as the reference outcome. The significance level was defined as two-sided *p* < 0.05 and all statistical analyses were performed using R (version 4.4.3) in RStudio (version 2026.10.0) [29].

## 3. Results

### 3.1. Metabolic Health-Related Dietary Index

Table 1 presents eight dietary components comprising the pre-diagnosis MHD index, along with their median (IQR) intake levels and assigned scoring direction. Components associated with adverse metabolic health including added sugars, glycemic load, saturated fat, and sodium, were scored in a positive direction with higher intake. On the other hand, fiber, fruit, vegetables, and whole grains scored in the negative direction with higher intake indicating a favorable metabolic health association.

### 3.2. Demographic Characteristics and Mortality Status

A total of 1497 men with prostate cancer were included in this analysis. Among those enrolled, 95 and 311 died from PCa and non-PCa causes respectively and 1091 were alive at follow-up. Participants who died from PCa were younger on average than those who died from non-PCa causes (*p* < 0.001). The distribution of race differed across mortality-status groups (*p* = 0.020). African American participants comprised 47.4% of those alive at follow-up, 46.9% of those who died from non-PCa causes, and 62.1% of those who died from PCa. Insurance status also differed across mortality-status groups (*p* = 0.002), as the proportion of participants who were insured was 91.8% among those alive at follow-up, 90.0% among those who died from non-PCa causes, and 81.1% among those who died from PCa. Similarly, previous PCa screening differed across mortality-status groups (*p* < 0.001), with previous screening reported by 90.2% of participants alive at follow-up, 81.7% of those who died from non-PCa causes, and 63.2% of those who died from PCa (Table 2).

MHD index was significantly different among the mortality outcome groups (*p* < 0.001), where the PCa death group scored a median of (0.43), indicating a more metabolically adverse dietary pattern relative to non-PCa death (0.08) and Alive (−0.01). The distribution of MHD index tertiles differed significantly by mortality status (*p* < 0.001). Among men who died from PCa, 50.5% were in the highest index tertile compared with 31.3% of men who were alive at follow-up, whereas 21.1% of PCa deaths and 35.7% of men alive were in the lowest tertile. WHR was categorized as low (<0.90), intermediate (0.90–<1.00), and high (≥1.00) and was significantly associated with mortality status (*p* < 0.001). A higher proportion of men who died of PCa had low WHR (22.6%) compared to men who died of non-PCa deaths (9.8%), while men who died due to PCa were less frequently classified in the intermediate (51.6% vs. 58.8%) and high (25.8% vs. 31.4%) WHR categories, PCa death to non-PCa death respectively. PCa aggressiveness was also associated with mortality status (*p* < 0.001), with a higher proportion of men who died due to PCa death having high aggressive PCa (86.3% vs. 33.1%).

The distribution of the MHD index is represented by mortality status, race, and PCa aggressiveness (Figure 2). Men who died of PCa death had a visibly higher median index score than those who died of non-PCa death or survived. Similarly, those with high aggressiveness also had a visibly higher median index score compared to those with low aggressiveness. No significant difference was found for race.

### 3.3. Adjusted Association Between MHD Index and Mortality Status

After adjustment for age at diagnosis, race, study site, smoking status, insurance status, previous PCa screening, and WHR, each 1-SD increase in the MHD index was associated with 38% higher odds of PCa-specific death relative to being alive at follow-up (aOR, 1.38; 95% CI, 1.10–1.73; *p* = 0.006). No association was observed with non-PCa death (aOR, 1.03; 95% CI, 0.89–1.19; *p* = 0.687). This is shown in Table 3. As a sensitivity analysis, we fitted an additional multivariable model that further adjusted for tumor aggressiveness, beyond the covariates included in the primary model. In this supplementary model, the association between the MHD index and PCa-specific death was attenuated but remained statistically significant (aOR = 1.26; 95% CI: 1.00–1.59; *p* = 0.048), whereas the association with non-PCa death remained null (aOR, 1.02; 95% CI, 0.88–1.18; *p* = 0.779). Additionally, the primary multivariable model was also run using tertiles of the MHD index, adjusted with the same covariates and the highest tertile (T3) showed a significant association with PCa death (aOR = 2.17, 95% CI: 1.21–3.87, *p* = 0.009). A statistically significant positive trend was observed across increasing tertiles of the MHD index for PCa-specific death (*p* for trend = 0.007), whereas the corresponding trend for non-PCa death was not statistically significant (*p* for trend = 0.304). This is shown in Table S2 and Figure S1. Age at diagnosis was positively associated with both mortality outcomes. Each one-year increase in age was associated with higher odds of PCa-specific death (aOR = 1.08, 95% CI: 1.05–1.11, *p* < 0.001) and non-PCa death (aOR = 1.11, 95% CI: 1.09–1.13, *p* < 0.001). The study site at UNC only showed a significant association to PCa death (*p* = 0.008). In contrast, race and WHR (all but group high - non-PCa death) were not significantly associated with either outcome (all *p* > 0.05; Table 3). High WHR category showed a significant association to non-PCa death (aOR = 1.81, 95% CI: 1.11–2.96, *p* = 0.018).

As shown in Figure 3, the association between the continuous MHD index and PCa death remains significant, though somewhat attenuated across all adjusted models. This suggests that part of the observed association may be attributed to covariates included in the fully adjusted model. As for non-PCa death, across all models, the association remains non-significant throughout and increasingly follows the same trend as PCa death, attenuating per additional covariate adjustments. Sensitivity analyses using the continuous MHD index are presented in Supplementary Table S5. In the primary adjusted model, a one-standard-deviation increase in the MHD index was associated with greater odds of PCa-specific death (aOR = 1.38; 95% CI: 1.10–1.73), but not non-PCa death (aOR = 1.03; 95% CI: 0.89–1.19). A formal comparison of regression coefficients indicated that these associations differed significantly (*p* = 0.021). Following additional adjustment for tumor aggressiveness, the association with PCa-specific death was attenuated but remained nominally statistically significant (aOR = 1.26; 95% CI: 1.00–1.59), while the difference between mortality outcomes was no longer statistically significant (*p* = 0.092). Bootstrap analysis using 500 resamples yielded estimates consistent with the primary model, with a median aOR of 1.39 (95% CI: 1.13–1.76) for PCa-specific death. Model diagnostics indicated satisfactory numerical stability and limited sensitivity to individual observations (Supplementary Table S4). The primary multinomial logistic regression model converged successfully, with no extreme fitted probabilities. Natural cubic spline analyses were conducted to assess potential nonlinear associations between the continuous MHD index and mortality status (Supplementary Figure S2). The association with PCa-specific death showed an overall increasing pattern across higher MHD index values, whereas the association with non-PCa death was relatively flat, with modest fluctuations around the null. However, the spline model did not provide a statistically significant improvement in fit compared with the linear model (likelihood ratio χ^2^ = 1.70, *df* = 4, *p* = 0.791).

## 4. Discussion

In this racially diverse cohort of men with PCa, a higher pre-diagnosis metabolic health-related dietary (MHD) index was significantly associated with increased odds of PCa death, but not non-PCa death. Each 1-SD increase in the MHD index was associated with 38% higher adjusted odds of PCa-specific death (aOR = 1.38, 95% CI 1.10–1.73; *p* = 0.006), whereas no corresponding association was observed for non-PCa death (aOR = 1.03, 95% CI 0.89–1.19; *p* = 0.687). The association with PCa-specific death was progressively attenuated across sequential adjustment models but remained evident after adjustment for demographic, lifestyle, healthcare-related, anthropometric, and tumor characteristics. These findings suggest that the combined characteristics of pre-diagnosis diet may be relevant to PCa prognosis.

Our findings are broadly consistent with previous evidence suggesting that overall dietary patterns may be more informative for PCa prognosis than individual foods or nutrients. In the Physicians’ Health Study, Yang et al. reported that greater adherence to a Western dietary pattern after diagnosis, characterized by greater consumption of processed and red meats, high-fat dairy products, and refined grain, was associated with higher PCa-specific mortality, whereas a prudent pattern characterized by vegetables, fruits, fish, legumes, and whole grains was associated with lower overall mortality [11]. The exposure period differs importantly from the present study, because our index reflects pre-diagnosis diet rather than post-diagnosis dietary behavior. Nevertheless, the direction of association is compatible with the broader hypothesis that dietary patterns characterized by poorer carbohydrate quality, higher saturated fat, and lower plant-food intake may be associated with less favorable outcomes among men with PCa.

Other studies provide additional, although not entirely consistent, support for this hypothesis. Higher saturated-fat intake has been associated with poorer outcomes in some cohorts of men with PCa. Among 926 men with nonmetastatic disease in the Physicians’ Health Study, Van Blarigan and colleagues reported higher PCa-specific mortality among men consuming a greater proportion of energy from saturated fat [34]. In a separate cohort of 4577 men with nonmetastatic PCa, replacing carbohydrate with vegetable fat was associated with lower risks of lethal PCa and all-cause mortality, although saturated fat itself was not significantly associated with lethal PCa in that analysis [35]. Earlier work also reported an association between higher saturated-fat consumption and PCa-specific mortality as men in the highest tertile of saturated-fat intake had approximately threefold higher risk of PCa-specific mortality than those in the lowest tertile (95% CI 1.3–7.7; *p* = 0.008) [36]. These findings illustrate both the potential relevance of dietary fat quality and the heterogeneity of the existing literature. Importantly, our index was not designed to isolate saturated fat or any other single nutrient, but rather to characterize the combined balance of multiple dietary attributes potentially relevant to metabolic health.

Biological pathways could plausibly link the dietary pattern captured by the index with PCa progression. Diets characterized by high glycemic burden and added sugars may contribute to repeated postprandial glucose and insulin responses, whereas dietary fat quality and low intake of fiber-rich plant foods may influence inflammatory and metabolic pathways [37]. These mechanisms are of interest in PCa because insulin-related signaling, adiposity, and chronic inflammation have been proposed as pathways linking metabolic health with tumor progression. Evidence from dietary inflammatory-pattern studies provides some support for this framework. In an Italian cohort, a higher Dietary Inflammatory Index was associated with PCa-specific mortality among men with higher Gleason scores, although not among men with lower-grade tumors [10]. In contrast, a recent Health Professionals Follow-up Study analysis of 4501 men with nonmetastatic PCa found no statistically significant associations between several post-diagnostic inflammatory, hyper-insulinemic, or insulin-resistance dietary/lifestyle indices and PCa-specific mortality [14].

This study has several strengths. Dietary intake was assessed using a comprehensive 144-item DHQ that captured usual intake during the 12 months preceding PCa diagnosis, allowing evaluation of pre-diagnosis dietary exposures. Rather than examining individual dietary components in isolation, we constructed a standardized MHD index integrating multiple dietary characteristics related to glycemic burden, fat quality, sodium intake, and consumption of fiber-rich plant foods. The analysis also distinguished PCa-specific deaths from deaths due to other causes, which is particularly relevant in PCa because competing causes of mortality are common. Finally, the association was examined using both the index as a continuous standardized measure and tertiles, with broadly consistent evidence of higher odds of PCa-specific death at higher index levels. Several limitations should also be considered. Dietary intake was self-reported and assessed retrospectively for the year preceding diagnosis, making the estimates susceptible to recall and measurement error. Diet was assessed at a single time point and therefore may not reflect dietary changes following diagnosis or treatment. Only 95 PCa-specific deaths occurred, limiting statistical precision, particularly in categorical and subgroup analyses. In addition, the non-PCa death category combined deaths from multiple causes with potentially different relationships to diet and metabolic health, which may have obscured cause-specific associations within this heterogeneous outcome. Although mortality status and cause-of-death classifications were available, the analytic dataset did not include individual dates of death or follow-up dates, precluding time-to-event analyses and estimation of mortality rates or hazard ratios. Consequently, multinomial logistic regression was used to examine associations with observed mortality status, and the resulting odds ratios should not be interpreted as estimates of mortality rates or hazards. Furthermore, although the models adjusted for several demographic, clinical, and lifestyle characteristics, residual confounding remains possible, particularly from unmeasured or incompletely characterized factors such as cancer treatment, comorbidities, socioeconomic circumstances, and post-diagnosis lifestyle changes. Finally, excluding participants with intermediate-aggressiveness PCa may limit the generalizability of the findings to the broader population of men diagnosed with PCa.

## 5. Conclusions

In conclusion, a more adverse pre-diagnosis dietary profile was associated with greater odds of PCa-specific death, but not death from other causes, suggesting a potentially disease-specific relationship with lethal PCa. Although adjustment for tumor aggressiveness and other clinical and lifestyle characteristics attenuated this association, the overall dietary pattern was associated with increased odds of PCa-specific mortality. These findings highlight the potential importance of the combined and cumulative influence of diet before diagnosis and future studies should also examine whether inflammatory markers and lipid measurements correspond to MHD index scores. This can support further evaluation of multidimensional dietary profiles as potentially modifiable factors associated with PCa progression and survival.

## Figures and Tables

**Figure 1:**
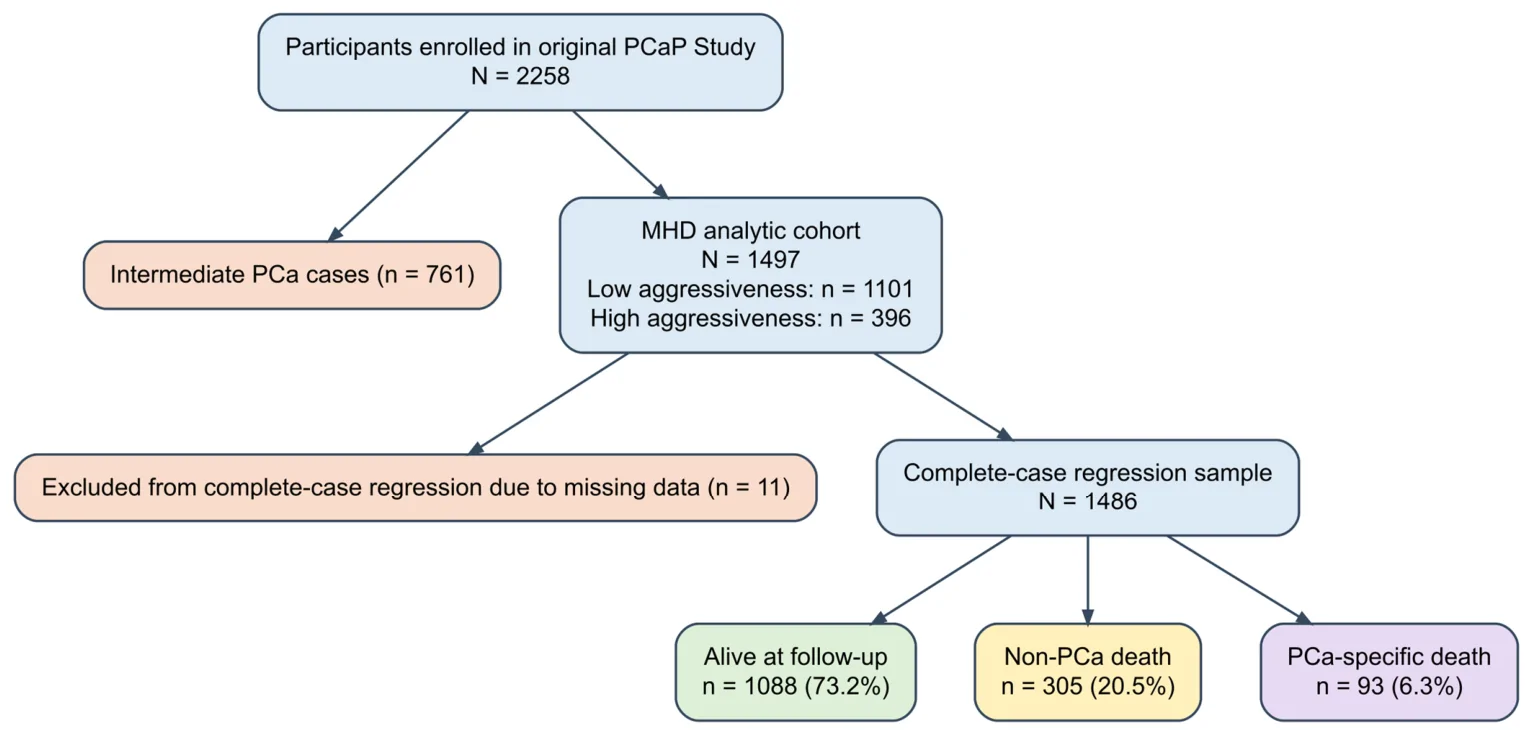
Participant flow diagram. Of the 2258 enrolled participants, 761 intermediate-aggressiveness cases were excluded, leaving 1497 participants in the analytic cohort. An additional 11 participants were excluded because of missing covariate data, resulting in a final complete-case regression sample of 1486 participants. Of these, 1088 were alive at follow-up, 305 died from causes other than prostate cancer, and 93 died from PCa.

**Figure 2:**
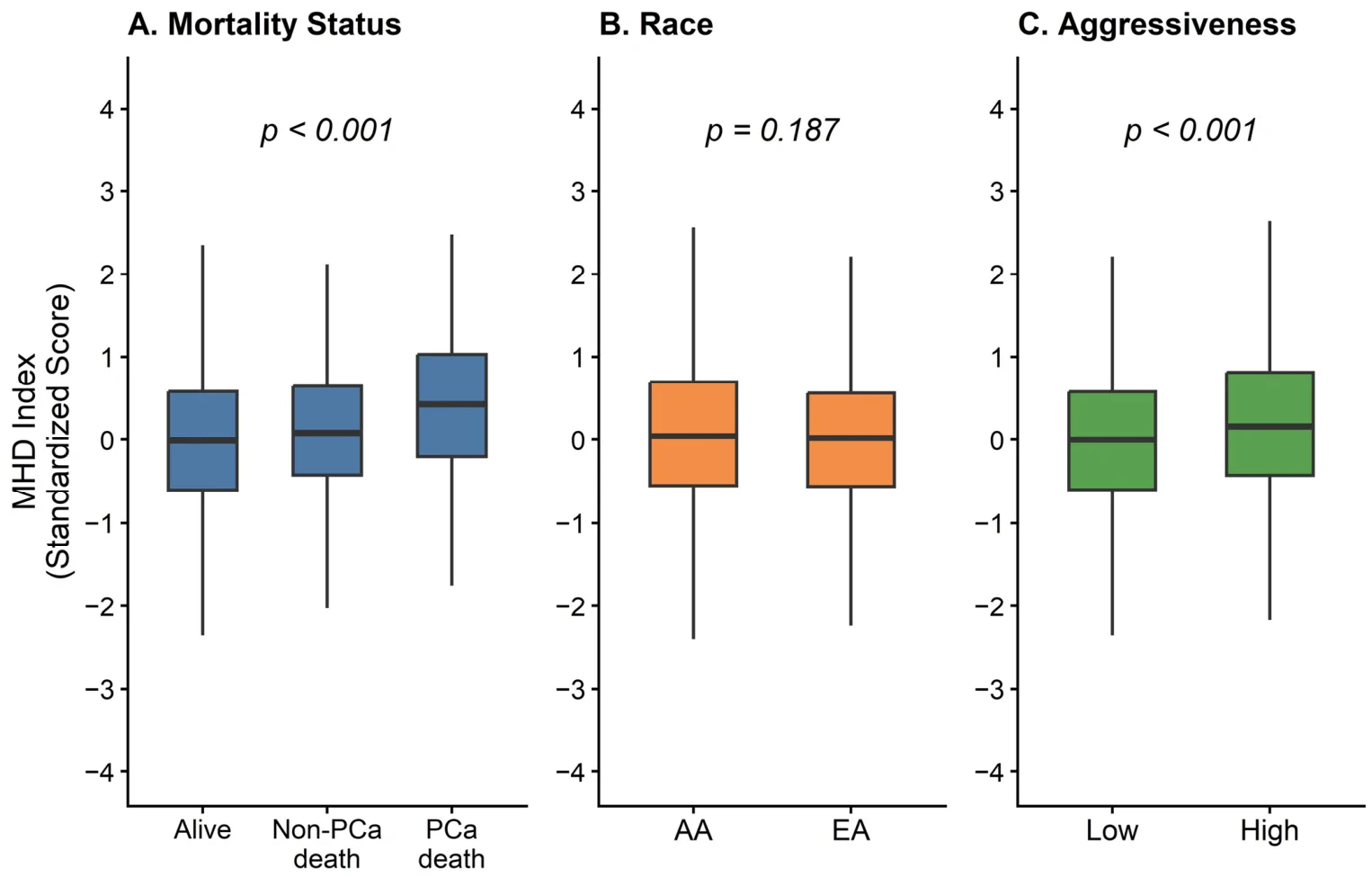
Distribution of the metabolic health-related dietary (MHD) index by mortality status, race, and tumor aggressiveness. (**A**) Among 1497 participants, mortality of 95 participants was from PCa-specific death, 311 due to non-PCa deaths, and 1091 were alive at follow-up. (**B**) Race included 722 African American (AA) and 775 European American (EA) participants. (**C**) PCa aggressiveness included 1101 low cases and 396 high aggressiveness cases.

**Figure 3:**
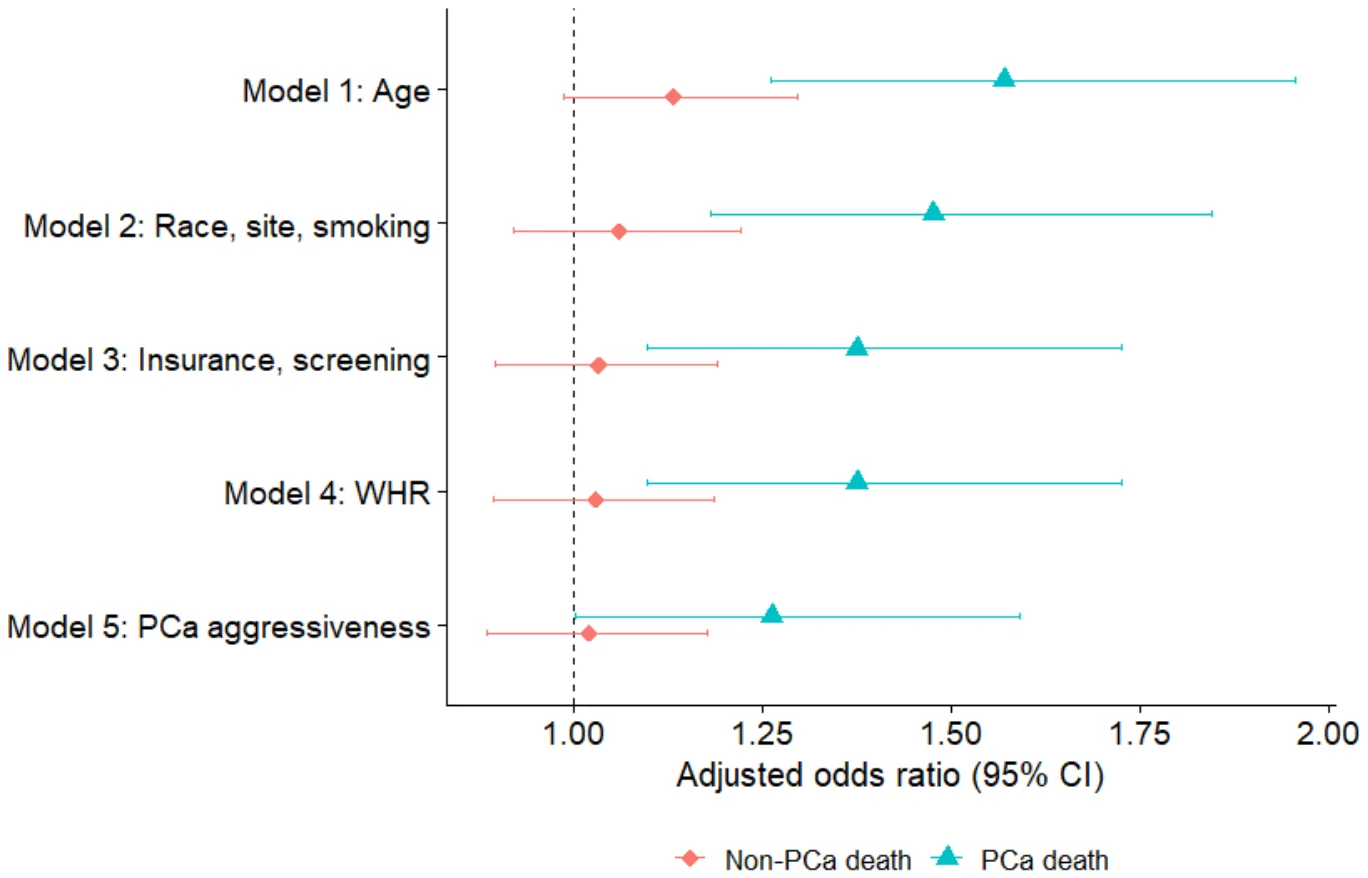
Association of the MHD index with mortality status across sequentially adjusted models. Points represent adjusted odds ratios (aORs) and horizontal bars represent 95% confidence intervals for non-PCa death and PCa death, relative to being alive at follow-up, per 1-SD increase in the MHD index. Tumor aggressiveness was considered separately and added in Model 5 as an additional adjustment because it was assessed at diagnosis and could potentially represent a downstream characteristic of pre-diagnosis dietary exposure.

**Table 1:** Components and scoring direction of the pre-diagnosis MHD index.

Component	Unit	Median (IQR)	Scoring Direction	Supporting Reference(s)
Added sugars	tsp/day	17.15 (10.22–29.27)	+	WHO, 2015; Lichtenstein et al., 2021 [30]
Glycemic load	units/day	133.38 (99.76–193.30)	+	Livesey et al., 2019 [31]
Saturated fat	% energy	10.73 (8.81–12.34)	+	WHO, 2023; Lichtenstein et al., 2021 [30]
Sodium	mg/day	3269 (2429–4460)	+	WHO, 2012
Fiber	g/day	19.67 (13.79–27.00)	−	Reynolds et al., 2019 [9]
Fruit	servings/day	2.42 (1.32–3.98)	−	Wang et al., 2014 [32]
Vegetables	servings/day	3.72 (2.38–5.45)	−	Wang et al., 2014 [32]
Whole grains	servings/day	0.94 (0.47–1.57)	−	Aune et al., 2016 [33]; Reynolds et al., 2019 [9]

Note: tsp = teaspoon. Values are median (interquartile range [IQR]) among 1497 participants. Each dietary component was standardized to a z-score before index construction. Added sugars, glycemic load, saturated fat, and sodium were positively scored (+), whereas fiber, fruit, vegetables, and whole grains were reverse-scored (−).

**Table 2:** Participant characteristics according to mortality status.

Characteristic	Overall (N = 1497)	Alive (N = 1091)	Non-PCa Death (N = 311)	PCa Death (N = 95)	*p*-Value
Age at diagnosis, years, mean (SD)	62.94 (7.94)	61.65 (7.60)	66.99 (7.68)	64.57 (7.99)	<0.001
MHD index, median (IQR), standardized score	0.03 (−0.56–0.63)	−0.01 (−0.60–0.59)	0.08 (−0.42–0.65)	0.43 (−0.20–1.03)	<0.001
MHD index tertile, n (%)					<0.001
Tertile 1 [ −4.06 to −0.33]	499 (33.3)	390 (35.7)	89 (28.6)	20 (21.1)	
T2 [>−0.33 to 0.41]	499 (33.3)	360 (33.0)	112 (36.0)	27 (28.4)	
T3 [>0.41 to 4.12]	499 (33.3)	341 (31.3)	110 (35.4)	48 (50.5)	
*Components of MHD index*					
Added sugars, tsp/day, median (IQR)	17.15 (10.22–29.27)	16.71 (10.13–27.25)	18.57 (10.94–31.58)	18.44 (9.39–37.38)	0.058
Glycemic load, units/day, median (IQR)	133.38 (99.76–193.30)	131.43 (99.14–187.37)	138.20 (102.11–205.15)	146.93 (91.70–206.31)	0.194
Saturated fat, % energy, median (IQR)	10.73 (8.81–12.34)	10.65 (8.77–12.29)	10.94 (8.81–12.35)	11.20 (9.10–12.68)	0.290
Sodium, mg/day, median (IQR)	3269 (2429–4460)	3248 (2432–4382)	3381 (2441–4565)	3394 (2323–4522)	0.682
Dietary fiber, g/day, median (IQR)	19.67 (13.79–27.00)	19.99 (13.90–26.73)	19.02 (13.79–27.31)	17.55 (11.73–27.46)	0.361
Fruit, servings/day, median (IQR)	2.42 (1.32–3.98)	2.43 (1.31–3.94)	2.54 (1.47–4.16)	1.96 (1.19–3.64)	0.097
Vegetables, servings/day, median (IQR)	3.72 (2.38–5.45)	3.74 (2.40–5.47)	3.73 (2.50–5.61)	3.28 (1.95–4.57)	0.166
Whole grains, servings/day, median (IQR)	0.94 (0.47–1.57)	0.99 (0.51–1.60)	0.84 (0.43–1.48)	0.59 (0.30–1.29)	<0.001
Race, n (%)					0.020
African American	722 (48.2%)	517 (47.4%)	146 (46.9%)	59 (62.1%)	
European American	775 (51.8%)	574 (52.6%)	165 (53.1%)	36 (37.9%)	
Study site, n (%)					0.146
LSU	791 (52.8%)	585 (53.6%)	165 (53.1%)	41 (43.2%)	
UNC	706 (47.2%)	506 (46.4%)	146 (46.9%)	54 (56.8%)	
Smoking status, n (%)					<0.001
Never	503 (33.7%)	412 (37.8%)	64 (20.6%)	27 (28.4%)	
Former	759 (50.8%)	542 (49.8%)	178 (57.4%)	39 (41.1%)	
Current	232 (15.5%)	135 (12.4%)	68 (21.9%)	29 (30.5%)	
Missing, n	3 (0.2%)	2 (0.2%)	1 (0.3%)	0	
PCa aggressiveness, n (%)					<0.001
Low	1101 (73.5%)	880 (80.7%)	208 (66.9%)	13 (13.7%)	
High	396 (26.5%)	211 (19.3%)	103 (33.1%)	82 (86.3%)	
Previous screening, n (%)					<0.001
No	199 (13.3%)	107 (9.8%)	57 (18.3%)	35 (36.8%)	
Yes	1298 (86.7%)	984 (90.2%)	254 (81.7%)	60 (63.2%)	
Insurance status, n (%)					0.002
Uninsured	138 (9.2%)	89 (8.2%)	31 (10.0%)	18 (18.9%)	
Insured	1359 (90.8%)	1002 (91.8%)	280 (90.0%)	77 (81.1%)	
Waist-to-hip ratio category, n (%)					<0.001
Low [<0.90]	219 (14.7%)	168 (15.4%)	30 (9.8%)	21 (22.6%)	
Intermediate [0.90–<1.00]	905 (60.8%)	677 (62.1%)	180 (58.8%)	48 (51.6%)	
High [≥1.00]	365 (24.5%)	245 (22.5%)	96 (31.4%)	24 (25.8%)	
Missing, n	8 (0.5%)	1 (0.1%)	5 (1.6%)	2 (2.1%)	

SD = standard deviation; IQR = interquartile range; MHD = metabolic health-related dietary; tsp = teaspoon; mg = milligram; g = gram.

**Table 3:** Multivariable associations of the metabolic health-related dietary (MHD) index and participant characteristics with mortality.

Variable	Non-PCa Death aOR (95% CI)	*p*-Value	PCa Death aOR (95% CI)	*p*-Value
MHD index, per 1-SD increase	1.03 (0.89–1.19)	0.687	1.38 (1.10–1.73)	0.006
Age (years)	1.11 (1.09–1.13)	<0.001	1.08 (1.05–1.11)	<0.001
Insurance status				
Insured	0.72 (0.43–1.18)	0.195	0.60 (0.31–1.16)	0.131
Previous screening				
Yes	0.50 (0.33–0.75)	<0.001	0.28 (0.16–0.47)	<0.001
Race				
European American	1.03 (0.78–1.37)	0.831	0.70 (0.43–1.13)	0.148
Study site				
UNC	1.17 (0.89–1.54)	0.260	1.84 (1.17–2.88)	0.008
Smoking status (ref = Never)				
Former	1.71 (1.23–2.39)	0.001	0.86 (0.51–1.46)	0.581
Current	3.66 (2.36–5.70)	<0.001	2.04 (1.09–3.83)	0.026
Waist-to-hip ratio category (ref = Low)				
Intermediate	1.36 (0.86–2.15)	0.182	0.65 (0.36–1.16)	0.147
High	1.81 (1.11–2.96)	0.018	0.91 (0.47–1.79)	0.792

Note: The fully adjusted model included 1486 participants with complete data for the exposure, mortality outcome, and included covariates.

## Data Availability

The data that support the findings of this study are available from the corresponding author, upon request. The North Carolina–Louisiana Prostate Cancer Project (PCaP) Executive Committee oversees the study data, which are not publicly available due to confidentiality restrictions established in the original participant consent forms.

## Supplementary Materials

The following supporting information can be downloaded at: https://www.mdpi.com/article/10.3390/nu18213703/s1, Table S1: Dietary components and scoring parameters used to construct the metabolic health-related dietary (MHD) index; Table S2: Association of MHD index tertiles and covariates with mortality status (Model without aggressiveness); Table S3: Association of MHD index tertiles and covariates with mortality status (Model with aggressiveness); Table S4: Model diagnostics; Table S5: Continuous MHD index sensitivity analyses; Figure S1: Association between tertiles of the pre-diagnosis metabolic health-related dietary (MHD) index and mortality status. Adjusted odds ratios (aORs) and 95% confidence intervals (CIs) are shown for non-PCa death and PCa-specific death relative to being alive at follow-up. The lowest MHD index tertile (T1) served as the reference category. Models were adjusted for age at diagnosis, race, study site, smoking status, insurance status, previous PCa screening, and WHR and tumor aggressiveness was not included in these models; Figure S2: Associations between the standardized metabolic health-related dietary (MHD) index and prostate cancer-specific and non-prostate cancer mortality using natural cubic spline regression. Models were adjusted for age at diagnosis, race, study site, smoking status, insurance status, previous prostate cancer screening, and waist-to-hip ratio category. The standardized MHD index value of zero was used as the reference (aOR = 1.0). Vertical dotted lines indicate the reference value, horizontal dashed lines indicate an aOR of 1.0, and rug marks represent the distribution of the standardized MHD index. The spline model did not significantly improve fit compared with the linear model (likelihood ratio χ^2^ = 1.70, *df* = 4, *p* = 0.791).
