# Supplementary material for: Pre-Diagnosis Metabolic Health-Related Dietary Index and Prostate Cancer–Specific Mortality
Source: Nutrients. 2026 Nov 8;18(21):3703. doi: 10.3390/nu18213703 (PMC13765276; doi:10.3390/nu18213703)
Supplement: Supplementary file 1 [file nutrients-18-03703-s001.zip › nutrients-4647273-supplementary.pdf]

Supplementary Materials

Table S1. Dietary components and scoring parameters used to construct the metabolic health-related dietary (MHD) index

Dietary component	Unit	Mean ± SD	Z-score calculation	Scoring weight
Added sugars	tsp/day	23.37 ± 21.22	$(X-23.37)/21.22$	+1
Glycemic load	units/day	157.97 ± 92.54	$(X-157.97)/92.54$	+1
Saturated fat	% energy	10.62 ± 2.75	$(X-10.62)/2.75$	+1
Sodium	mg/day	3,679.23 ± 1,991.22	$(X-3679.23)/1991.22$	+1
Dietary fiber	g/day	21.84 ± 11.54	$(X-21.84)/11.54$	−1
Fruit	servings/day	3.01 ± 2.48	$(X-3.01)/2.48$	−1
Vegetables	servings/day	4.22 ± 2.49	$(X-4.22)/2.49$	−1
Whole grains	servings/day	1.14 ± 0.93	$(X-1.14)/0.93$	−1

SD, standard deviation; X, participant-specific dietary intake; tsp, teaspoon.

Table S2. Association of MHD index tertiles and covariates with mortality status (Model without aggressiveness)

Variable	Non-PCa death aOR (95% CI)	P-value	PCa death aOR (95% CI)	P-value
MHD index tertile (ref = T1)				
T2	1.38 (0.99–1.93)	0.061	1.53 (0.82–2.83)	0.179
T3	1.19 (0.85–1.68)	0.313	2.17 (1.21–3.87)	0.009
P for trend		0.301		0.007
Age (years)	1.11 (1.09–1.13)	<0.001	1.08 (1.04–1.11)	<0.001
Insurance status				
Insured	0.72 (0.44–1.19)	0.197	0.62 (0.32–1.20)	0.157
Previous screening				
Yes	0.50 (0.33–0.75)	<0.001	0.28 (0.17–0.48)	<0.001
Race				
European American	1.02 (0.77–1.36)	0.872	0.69 (0.43–1.12)	0.131
Study site				

Variable	Non-PCa death aOR (95% CI)	P-value	PCa death aOR (95% CI)	P-value
UNC	1.17 (0.89–1.54)	0.251	1.78 (1.14–2.79)	0.011
Smoking status (ref = Never)				
Former	1.72 (1.24–2.40)	0.001	0.88 (0.52–1.49)	0.629
Current	3.64 (2.34–5.65)	<0.001	2.11 (1.13–3.94)	0.019
Waist-to-hip ratio category (ref = Low) [<0.90]				
Intermediate [0.90–<1.00]	1.35 (0.86–2.14)	0.194	0.64 (0.36–1.14)	0.128
High [≥1.00]	1.80 (1.10–2.95)	0.020	0.89 (0.46–1.74)	0.730

Table S3. Association of MHD index tertiles and covariates with mortality status (Model with aggressiveness)

Characteristic	Non-PCa death vs Alive, aOR (95% CI)	P-value	PCa death vs Alive, aOR (95% CI)	P-value
MHD index tertile (Ref= Tertile 1)				
T2	1.35 (0.96–1.89)	0.081	1.36 (0.71–2.62)	0.359
T3	1.17 (0.83–1.66)	0.362	1.88 (1.01–3.48)	0.046
P for trend		0.351		0.038
Age at diagnosis, per year	1.11 (1.08–1.13)	<0.001	1.06 (1.02–1.09)	0.001
Tumor aggressiveness				
High	1.49 (1.10–2.03)	0.011	20.31 (10.90–37.86)	<0.001
Insurance status				
Insured	0.74 (0.45–1.22)	0.239	0.72 (0.35–1.48)	0.375
Previous prostate cancer screening				
Yes	0.52 (0.34–0.79)	0.002	0.37 (0.21–0.66)	<0.001
Race				
European American	1.06 (0.79–1.41)	0.712	0.90 (0.53–1.53)	0.695
Study site				
UNC	1.19 (0.91–1.57)	0.210	2.03 (1.25–3.28)	0.004
Smoking status (Ref= Never)				
Former	1.69 (1.21–2.36)	0.002	0.76 (0.43–1.34)	0.341

Characteristic	Non-PCa death vs Alive, aOR (95% CI)	P-value	PCa death vs Alive, aOR (95% CI)	P-value
Current	3.45 (2.22–5.37)	<0.001	1.64 (0.82–3.28)	0.160
Waist-to-hip ratio				
Low [<0.90]	1.00 (Reference)	—	1.00 (Reference)	—
Intermediate [0.90–<1.00]	1.39 (0.88–2.19)	0.163	0.62 (0.33–1.18)	0.146
High [≥1.00]	1.75 (1.07–2.88)	0.027	0.68 (0.32–1.36)	0.255

Note: Multinomial logistic regression was used with alive at follow-up as the reference outcome. The fully adjusted model included 1,486 participants with complete data

Table S4. Model diagnostics

Diagnostic	Result
Primary model convergence	Successful
Minimum Hessian eigenvalue	0.932
Condition number before age standardization	1,043,343
Condition number after age standardization	310.46
Extreme fitted probabilities	None
Successful leave-one-out refits	1,486/1,486
PCa-specific MHD aOR range after individual deletion	1.34–1.43
Maximum relative change in PCa-specific MHD aOR	3.77%
Successful bootstrap refits	500/500
Bootstrap PCa-specific aOR, median (95% percentile CI)	1.39 (1.13–1.76)

Table S5. Continuous MHD index sensitivity analyses

Model	Non-PCa death aOR (95% CI)	PCa-specific death aOR (95% CI)	P for coefficient difference
Primary adjusted model	1.03 (0.89–1.19)	1.38 (1.10–1.73)	0.021
Additionally adjusted for tumor aggressiveness	1.02 (0.89–1.18)	1.26 (1.00–1.59)	0.092
Bootstrap median, 500 resamples	1.03 (0.89–1.21)	1.39 (1.13–1.76)	—

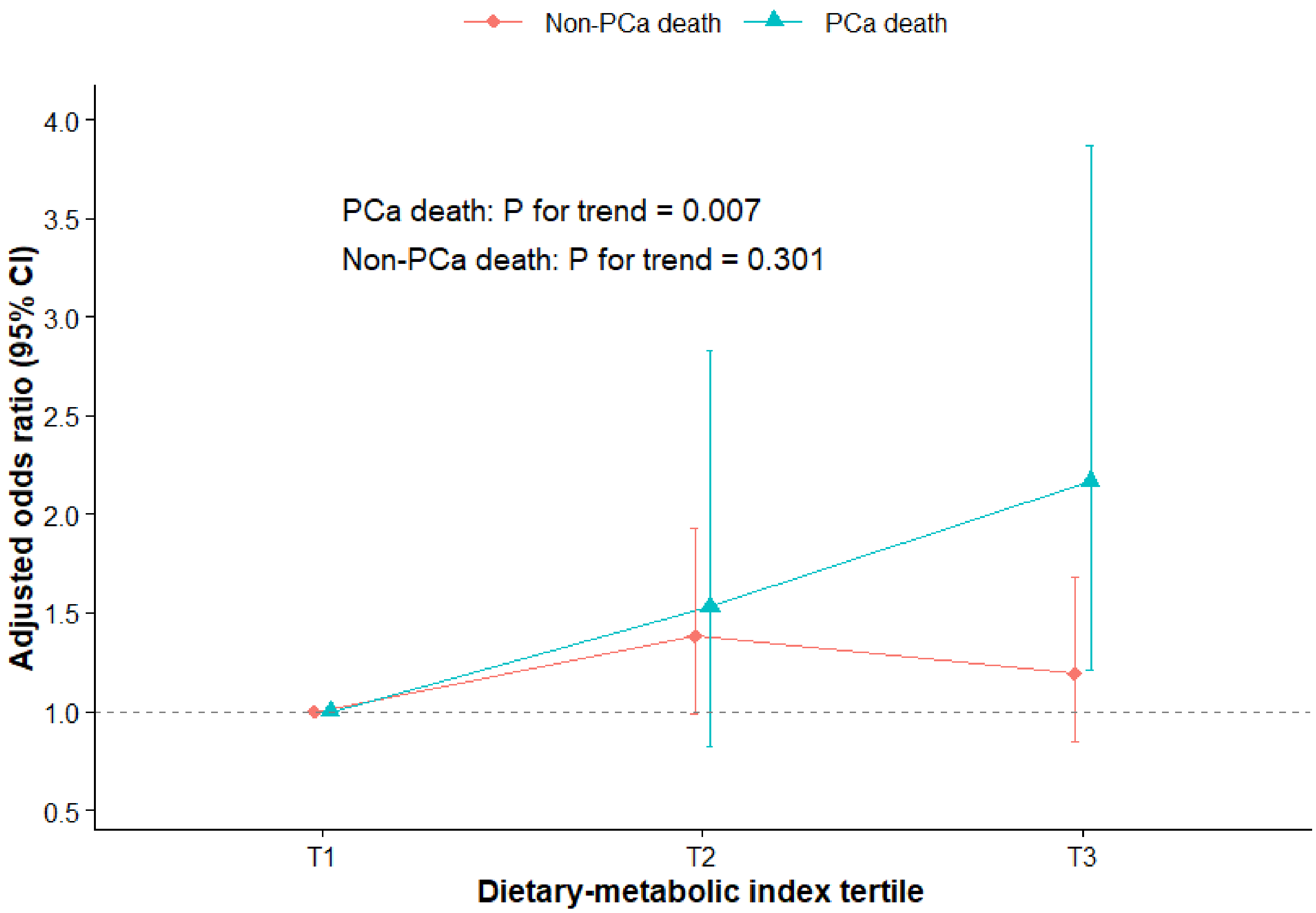

Figure S1. Association between tertiles of the pre-diagnosis metabolic health-related dietary (MHD) index and mortality status. Adjusted odds ratios (aORs) and 95% confidence intervals (CIs) are shown for non-PCa death and PCa-specific death relative to being alive at follow-up. The lowest MHD index tertile (T1) served as the reference category. Models were adjusted for age at diagnosis, race, study site, smoking status, insurance status, previous PCa screening, and WHR and tumor aggressiveness was not included in these models.

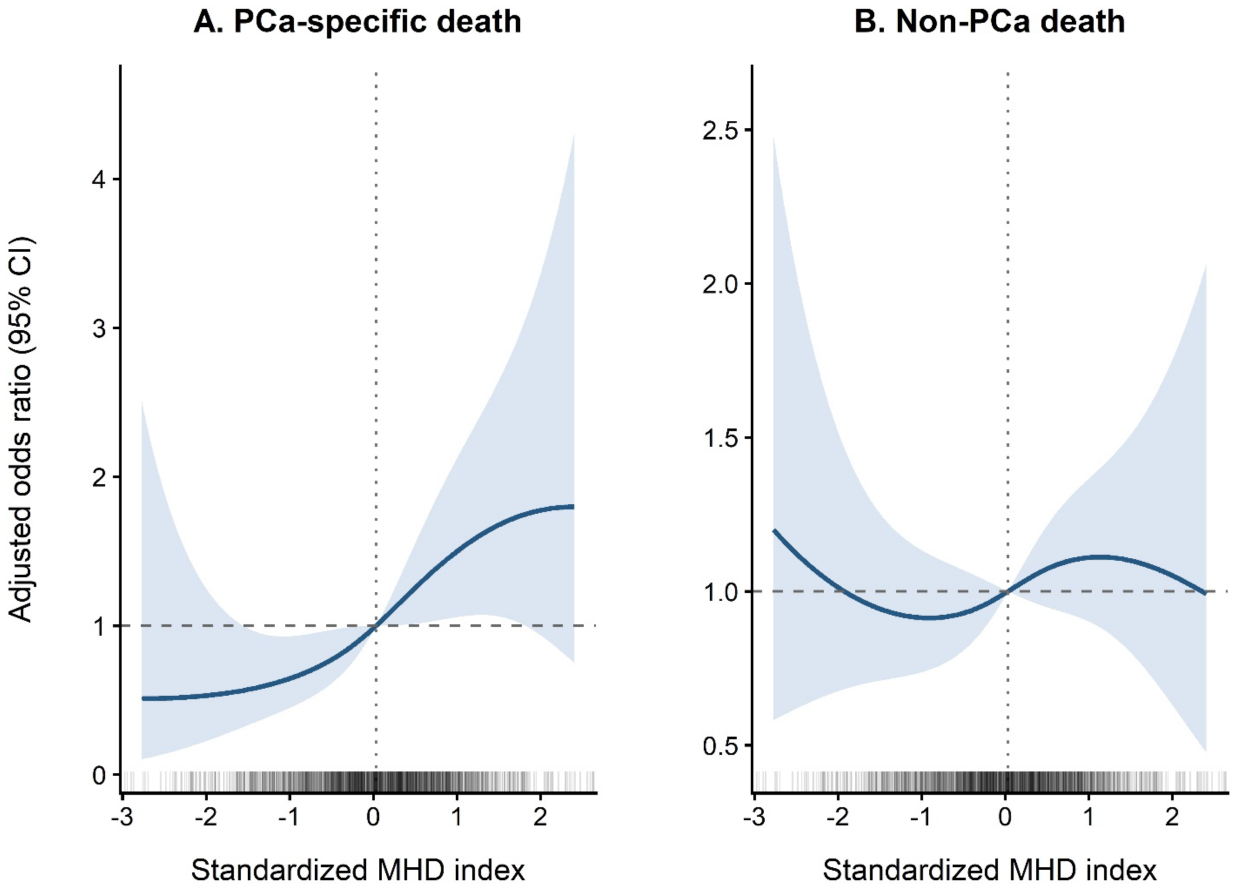

Figure S2. Associations between the standardized metabolic health-related dietary (MHD) index and prostate cancer-specific and non-prostate cancer mortality using natural cubic spline regression. Models were adjusted for age at diagnosis, race, study site, smoking status, insurance status, previous prostate cancer screening, and waist-to-hip ratio category. The standardized MHD index value of zero was used as the reference (aOR = 1.0). Vertical dotted lines indicate the reference value, horizontal dashed lines indicate an aOR of 1.0, and rug marks represent the distribution of the standardized MHD index. The spline model did not significantly improve fit compared with the linear model (likelihood ratio $\chi^2 = 1.70$, df = 4, $p = 0.791$).
